# Therapeutic efficacy of azathioprine in addition to prednisone-based regimens as first-line chronic graft-versus-host disease treatment

**DOI:** 10.1038/s41409-017-0025-0

**Published:** 2017-12-15

**Authors:** Jieun Uhm, Elizabeth Shin, Fotios V. Michelis, Santhosh Thyagu, Auro Viswabandya, Jeffrey H. Lipton, Hans A. Messner, Dennis (Dong hwan) Kim

**Affiliations:** 1 Allogeneic Blood And Marrow Transplant Program, Division of Medical Oncology and Hematology, Princess Margaret Cancer Centre, Department of Medicine, University of Toronto, 610 University Ave. Toronto, Ontario, Canada M5G2M9; 2 Hematology and Oncology, Department of Internal Medicine, Hanyang University Seoul Hospital, College of Medicine, Hanyang University, Seoul, Korea; 3 School of Medicine, University of Toronto, Toronto, Canada

Chronic graft-versus-host disease (cGVHD) is a major complication of allogeneic hematopoietic stem cell transplantation (allo-HCT) that is related to higher mortality and morbidity [1, 2]. Glucocorticoids has been the mainstay of the treatment for cGVHD, while it also has been widely used to treat the variety of autoimmune diseases as the combination with other immunosuppressive agents including azathioprine (AZP) to reduce long-term complications of glucocorticoids such as diabetes mellitus, iatrogenic Cushing’s syndrome, avascular necrosis of joints and osteoporosis, etc. [3–6].

Although a previous clinical trial suggested that prednisone (PRD) based regimen plus AZP (PRD + AZP) resulted in worse survival than PRD-based regimen in a standard risk group of cGVHD patients due to higher non-relapse (infection-related) mortality (NRM) [7], the therapeutic efficacy of AZP might deserve to be looked at again because there have been advances in the allo-HCT field for over the last decades, including significant improvement in supportive care such as infectious prophylaxis and treatment, as well as in evaluating cGVHD systematically. The National Institutes of Health (NIH) first proposed consensus criteria for the diagnosis of cGVHD, and tools for scoring cGVHD organ involvement and assessing overall severity in 2005, which are now widely used in clinical practice [8, 9]. In addition, a new statistical endpoint for evaluating the efficacy of cGVHD treatment, i.e. failure free survival (FFS), has been introduced and suggested to be a potential surrogate of overall survival (OS) for cGVHD treatment [10, 11]. Therefore, we retrospectively reviewed 668 consecutive patients who underwent allo-HCT between 2004 and 2012 at Princess Margaret Cancer Centre, Toronto, Canada in order to compare the efficacy of PRD + AZP and PRD-based regimens with respect to FFS as well as OS, NRM, and the incidence of relapse.

Chronic GVHD was defined, reclassified and graded by the NIH consensus criteria [8]. Among 313 patients with redefined cGVHD, we then identified 240 patients who received PRD or PRD + AZP as first line treatment for cGVHD. Late onset acute GVHD was excluded from the analysis.

The FFS was defined as time from the initiation of frontline treatment for cGVHD to treatment failure (TF), NRM or relapse of disease. TF was defined as initiation of the next line of IST for cGVHD [11] or an escalation of the dose of PRD to ≥1 mg/kg/day regardless of the target organ. OS and FFS were calculated by the Kaplan–Meier method and compared using the log rank test. The cumulative incidences of NRM, disease relapse, and the TF rate (TFR) for front line cGVHD treatment were estimated considering competing risks, with disease relapse, NRM and TFR considered as mutually-competing risks.

The transplant-related characteristics were analyzed to compare the PRD and PRD + AZP groups using Pearson’s Χ^2^ or Fisher’s exact test. The univariate and multivariate analyses performed to compare OS, NRM, relapse incidence, and FFS between two treatment groups. OS and FFS were compared using the log rank test. Univariate analyses for incidence with competing risks were performed by Gray’s method. Cox proportional hazard regression model was used for multivariate analysis of survivals.

Since the characteristics of cGVHD of two treatment groups were imbalanced (Table 1), we performed a propensity score matching (PSM) analysis as a case-control study in order to adjust the potential confounding effects of the clinical features of cGVHD on treatment outcome. The clinical variables included in the propensity score calculations were global score (GS) by the NIH consensus criteria, the classification of the cGVHD (classical or overlap syndrome), age, gender, duration from allo-HCT to initiation of cGVHD treatment, performance status (PS), progressive type onset (PTO) of cGVHD, thrombocytopenia and organs involved cGVHD per skin, gastrointestinal track, liver, lung, and musculoskeletal system. A total of 74 case-control pairs were identified with <0.1 of a difference in propensity score.

**Table 1:** Characteristics of patients and chronic GVHD

	Whole cohort				Propensity score matching analysis cohort			
	All	Prednisone alone	Prednisone and Azathioprine	*p*-value	All	Prednisone alone	Prednisone and Azathioprine	*p*-value
	(%, *n*=240)	(%, *n*=142)	(%, *n*=98)		(%, *n*=148)	(%, *n*=74)	(%, *n*=74)	
Median age at transplant, year (range)	50 (19–70)	50 (19–68)	51 (19–70)		52 (20–70)	52 (21–69)	52 (20–70)	
Gender, no. (%)								
Male	137 (57.1)	79 (55.6)	58 (59.2)	0.598	83 (56)	41 (55)	42 (57)	0.868
Female	103 (42.9)	63 (44.4)	40 (40.8)		65 (44)	33 (46)	32 (43)	
Gender mismatch, no. (%)								
Female to male	53 (22.1)	31 (21.8)	22 (22.4)	1.000	31 (21)	14 (19)	17 (23)	0.545
Other	187 (77.9)	111 (78.2)	76 (77.6)		117 (79)	60 (81)	57 (77)	
Disease, no. (%)								
AML	104 (43.3)	60 (42.3)	44 (44.9)	0.760	64 (43)	33 (45)	31 (42)	0.791
ALL	22 (9.1)	11 (7.7)	11 (11.2)		13 (9)	8 (11)	5 (7)	
MDS	23 (9.6)	13 (9.2)	10 (10.2)		16 (11)	7 (10)	9 (12)	
CML	17 (7.1)	13 (9.2)	4 (4.1)		9 (6)	6 (8)	3 (4)	
CLL	21 (8.8)	14 (9.9)	7 (7.1)		14 (10)	7 (10)	7 (10)	
MF/MPD	21 (8.7)	12 (8.5)	9 (9.2)		11 (7)	4 (5)	7 (10)	
Malignant lymphoma	27 (11.3)	15 (10.6)	12 (12.2)		20 (14)	9 (12)	11 (15)	
AA	4 (1.7)	3 (2.1)	1 (1.0)		1 (1)	0 (0)	1 (1)	
MM	1 (0.4)	1 (0.7)	0 (0)		0 (0)	0 (0)	0 (0)	
Intensity of conditioning regimen, no. (%)								
Myeloablative	154 (64.2)	93 (65.5)	61 (62.2)	0.681	90 (61)	47 (64)	43 (58)	0.501
Non-myeloablative	86 (35.8)	49 (34.5)	37 (37.8)		58 (39)	27 (37)	31 (42)	
HLA and donor type, no. (%)								
Related	146 (60.8)	84 (59.1)	62 (62.2)	0.489	96 (65)	46 (62)	50 (68)	0.591
Unrelated	86 (35.8)	54 (38)	32 (32.7		46 (31)	25 (34)	21 (28)	
Missing	8 (3.3)	4 (2.8)	4 (4.1)		6 (4)	3 (4)	3 (4)	
Stem cell source, no. (%)								
Bone marrow	19 (7.9)	12 (8.5)	7 (7.1)	0.811	8 (5)	4 (5)	4 (5)	1
Peripheral blood	221 (92.1)	130 (91.5)	91 (92.9)		140 (95)	70 (95)	70 (95)	
T-cell depletion, no. (%)	46 (19.2)	33 (23.2)	13 (13.3)	0.054	24 (16)	16 (22)	8 (11)	0.074
Any grade of acute GVHD	181 (78.9)	113 (81.9)	68 (73.9)	0.148	106 (72)	57 (77)	49 (66)	0.149
Median onset of cGVHD, Day (95% CI)	140 (131–149)	132 (123–141)	160 (140–180)	<0.001	140 (128–151)	140 (130–149)	152 (127–176)	0.863
Classification of NIH cGVHD								
Classical	87 (36.2)	48 (33.8)	39 (39.8)	0.418	51 (34.5)	26 (35.1)	25 (33.8)	0.863
Overlap syndrome	153 (63.8)	94 (66.2)	59 (60.2)		97 (65.5)	48 (64.9)	49 (65.5)	
Global score of cGVHD at treatment								
Mild	24 (10.0)	16 (11.3)	8 (8.2)	<0.001	14 (9.5)	6 (8.1)	8 (10.8)	0.319
Moderate	173 (72.1)	89 (62.7)	84 (85.7)		122 (82.5)	59 (79.7)	62 (83.8)	
Severe	43 (17.9)	37 (26.1)	6 (6.1)		12 (8.1)	9 (12.2)	4 (5.4)	
Organs involved in cGVHD								
Skin	155 (64.6)	88 (62.0)	67 (68.4)	0.108	101 (68)	54 (73)	47 (64)	0.219
Mouth	116 (48.3)	66 (46.5)	50 (51.0)	0.359	72 (49)	33 (45)	39 (53)	0.341
Eyes	77 (32.1)	45 (31.7)	32 (32.7)	0.889	46 (31)	21 (28)	25 (34)	0.477
Gastrointestinal tract	50 (20.8)	36 (25.4)	14 (14.3)	0.052	22 (15)	12 (16)	10 (14)	0.644
Liver	166 (69.2)	94 (66.2)	72 (73.5)	0.202	109 (74)	55 (74)	54 (73)	0.604
Lung	21 (8.8)	18 (12.7)	3 (3.1)	0.010	5 (3)	2 (3)	3 (4)	0.649
Musculoskeletal system	13 (5.4)	6 (4.2)	7 (7.1)	0.326	7 (5)	4 (5)	3 (4)	0.699
Others	7 (2.9)	4 (2.8)	3 (3.1)	0.912	5 (3)	3 (4)	2 (3)	0.649
No. of organs involved								
1–2	122 (50.8)	76 (53.5)	46 (46.9)	0.523	79 (53.4)	41 (55.4)	38 (51.4)	0.789
3	74 (30.8)	40 (28.2)	34 (34.7)		44 (20.7)	22 (29.7)	22 (29.7)	
4 or more	44 (18.3)	26 (18.3)	18 (18.4)		25 (16.9)	1 (14.9)	14 (18.9)	
Progressive type onset	25 (10.5)	22 (15.5)	3 (3.1)	0.002	7 (4.7)	4 (5.4)	3 (4.1)	1
Extensive skin involvement	84 (35.4)	51 (35.9)	33 (34.7)	0.468	59 (41)	31 (42)	28 (39)	0.576
ECOG performance status								
0–1	185 (77.1)	101 (71.1)	84 (85.7)	0.008	129 (87.2)	65 (87.8)	64 (86.5)	0.806
2 or higher	55 (22.9)	41 (28.9)	14 (14.2)		19 (12.8)	9 (12.2)	10 (13.5)	
Thrombocytopenia	67 (27.9)	49 (34.5)	18 (18.4)	0.008	34 (23.0)	18 (24.3)	16 (21.6)	0.696
Eosinophilia	86 (35.8)	49 (34.5)	18 (18.4)	0.020	62 (42)	31 (42)	31 (42)	1.000
Lymphopenia	153 (64.0)	98 (68.5)	55 (56.1)	0.021	89 (61)	45 (62)	44 (60)	0.613
Calcineurin inhibitors in addition to PRD or PRD + AZP	149 (62.1)	99 (69.7)	50 (56.1)	0.003	87 (58.8)	45 (60.8)	42 (56.8)	0.616

*AML* acute myeloid leukemia, *ALL* acute lymphoblastic leukemia, *MDS* myelodysplastic syndrome, *CML* chronic myelogenous leukemia, *MF* myelofibrosis, *MPD* myeloproliferative disorder, *CLL* chronic lymphocytic leukemia, *AA* aplastic anemia, *MM* multiple myeloma, *HLA* human leukocyte antigen, *GVHD* graft-versus-host disease, *cGVHD* chronic GVHD, *95% CI* 95% confidence interval

Of the 240 patients included in the analysis, 154 (64.2%) received myeloablative conditioning (MAC) and 86 (35.8%) reduced-intensity conditioning (RIC) (Table 1). There were no significant differences in pretransplant characteristics between the PRD and PRD + AZP groups except for T-cell depletion (TCD); 33 patients (23.2%) in the PRD group and 13 (13.3%) in the PRD + AZP group underwent T-cell depletion (*p* = 0.054). The imbalanced characteristics of cGVHD were observed between the 2 groups, including longer duration from HCT to diagnosis of cGVHD (*p* < 0.001) in the PRD + AZP group; also fewer patients with severe cGVHD (*p* < 0.001), fewer with PTO (*p* = 0.002), fewer with thrombocytopenia (*p* = 0.008) and better PS (*p* = 0.008).

With a follow-up duration of 43.6 months among survivors, 2-year FFS, TFR, NRM, and relapse incidence were 24.7% (95% confidence interval (CI), 19.1–30.8%), 57.5% (50.8–64.0%), 7.5% (4.5–11.5%), and 10.1% (6.5–14.5%), respectively. The PRD + AZP group had a higher FFS rate at 2 years (36.4% [26.2–46.6%]) than the PRD group (16.8% [10.8–23.9%], *p* < 0.001) (Fig. 1a) and a lower incidence of TFR at 2 years (52% [40.8–62.0%] versus 61.5% [52.5–69.3%], *p* = 0.050). In addition, it had a lower NRM rate at 2 years (3.4% [0.9–8.85] versus 10.5% [6–16.5%], *p* = 0.050). There was no difference between the groups in the cumulative incidence of relapse at 2 years; 8.3% (3.6–15.5%, *p* = 0.507) in PRD + AZP group and 11.3% (6.5–17.4%) in PRD group.

**Fig. 1:**
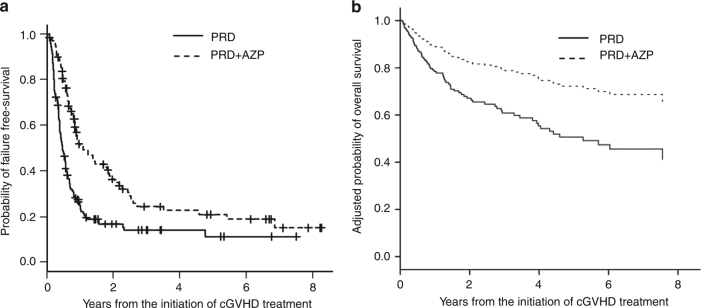
Survivals and the treatment failure (*n* = 240). **a** Failure-free survival comparing the prednisone and prednisone/azathioprine groups. **b** Adjusted overall survival comparing the prednisone and prednisone/azathioprine groups considering the severities of chronic graft-versus-host disease and performance status

Severity by the NIH consensus criteria was well-correlated with FFS. The FFS rate at 2 years was 62.2% (39.9–78.3%) in mild, 20.5% (14.2–27.7%) in moderate, and 16.9% (7.5–29.6%) in severe cGVHD (*p* < 0.001). Patients with mild cGVHD had a lower TFR (29.2% [12.6–48.1%]) at 2 years than those with moderate/severe cGVHD (61.4% [54–68%], *p* = 0.008). Severity by the NIH consensus criteria does not correlate with the cumulative incidence of NRM (*p* = 0.538) or relapse (*p* = 0.826). None of the factors associated with FFS or cumulative incidences of TFR and NRM were correlated with relapse rate.

OS at 2 years was 71.6% (64.6–77.4%); PRD + AZP group showed better survival compared to the PRD group (OS at 2 years; 82.1% [71–89.2%] versus 64.8% [55.4–72.8%], *p* < 0.001). And the severities of cGVHD and PS correlated well with 2-year OS (*p* = 0.004 and *p* < 0.001, respectively). The adjusted OS for PRD and PRD + AZP groups demonstrated statistical significance considering the severities of cGVHD and PS (HR for PRD group 2.09 [1.22–3.58], *p* = 0.007) (Fig. 1b).

Univariate analysis for FFS identified several risk factors associated with worse FFS including moderate/severe cGVHD (median FFS (months); 55.9 versus 7.6, *p* = 0.001), ECOG PS ≥ 2 (9 versus 5.4, *p* = 0.003), thrombocytopenia (6.5 versus 6.2, *p* = 0.05), PTO (8.6 versus 2.7, *p* = 0.001), and PRD group (13.2 versus 5.6, *p* < 0.001). Multivariate analysis confirmed that moderate/severe cGVHD (hazard ratio [HR] 3.10, *p* < 0.001), PTO (HR 2.21, *p* = 0.001) and PRD (versus PRD + AZP) as the first-line treatment regimen (HR 2.12, *p* < 0.001) were risk factors for worse FFS.

After PSM, the characteristics of cGVHD were well-balanced in the two groups (Table 1). The PSM analysis confirmed the findings of superior outcomes in the PRD + AZP group. Two-year FFS was significantly better in the PRD + AZP (36.4%) than the PRD group (16.8%, *p* < 0.001). The cumulative incidence of TFR for frontline treatment at 2 years was also lower in the PRD + AZP group (52.4% versus 70.1%, *p* = 0.013). There were no significant differences in NRM or relapse rate at 2 years, but a trend towards longer OS was again observed in the PRD + AZP group of the PSM cohort (85.3% [72.6–92.4%] at 2 years in PRD + AZA group versus 75.9% [63.1–84.8%] in PRD group, *p* = 0.066).

When confined to the same severity level according to the NIH consensus criteria, there was also a trend towards longer FFS in the PRD + AZP group: the favorable effect of PRD + AZP was statistically significant in the subgroup with moderate grade of cGVHD [FFS at 2 years (%); 30.5 versus 9.1, *p* = 0.001], but not in the mild and severe grades. Similar results were obtained for the cumulative incidence of TFR of frontline treatment at 2 years among the patients with moderate cGVHD; 56.2% (41.6–68.6%) in the PRD + AZP group and 71.4% (46.8–81.7%) in the PRD group (*p* = 0.035).

In addition, it was found that tapering of PRD dose < 0.5 mg/kg/day was more successful in the PRD + AZP group than in the PRD group: the cumulative incidence of PRD < 0.5 mg/kg/day at 6 months was 90.5% in the PRD + AZP group and 75.8% in PRD group (*p* = 0.018).

Although PSM analysis performed to overcome and control the imbalance of patients’ characteristics between PRD and PRD + AZP groups, the results of this study should be interpreted with caution given the nature of the retrospective analysis of this study, which would be weak evidence to support the role of AZP in cGVHD treatment compared the previous trial [7]. However, AZP added to a PRD-based regimen as the first-line treatment for cGVHD seems to improve FFS and may have a role as a steroid-sparing agent in the modern allo-HCT era. Since two thirds of the patients who required PRD-based treatment for cGVHD experienced the TF at 2 years, a better treatment strategy would be required. AZP could be worth reconsidered as a relevant option for a steroid sparing agent in cGVHD treatment.

## References

[1] Lee SJ, Klein JP, Barrett AJ, Ringden O, Antin JH, Cahn JY (2002). Severity of chronic graft-versus-host disease: association with treatment-related mortality and relapse. Blood.

[2] Sutherland HJ, Fyles GM, Adams G, Hao Y, Lipton JH, Minden MD (1997). Quality of life following bone marrow transplantation: a comparison of patient reports with population norms. Bone Marrow Transplant..

[3] Choy EH, Isenberg DA (2002). Treatment of dermatomyositis and polymyositis. Rheumatology..

[4] Hahn BH, McMahon MA, Wilkinson A, Wallace WD, Daikh DI, Fitzgerald JD (2012). American College of Rheumatology guidelines for screening, treatment, and management of lupus nephritis. Arthritis Care Res..

[5] Kirk AP, Lennard-Jones JE (1982). Controlled trial of azathioprine in chronic ulcerative colitis. Br Med J. (Clin ResEd).

[6] Meriggioli MN, Sanders DB (2009). Autoimmune myasthenia gravis: emerging clinical and biological heterogeneity. Lancet Neurol..

[7] Sullivan KM, Witherspoon RP, Storb R, Weiden P, Flournoy N, Dahlberg S (1988). Prednisone and azathioprine compared with prednisone and placebo for treatment of chronic graft-v-host disease: prognostic influence of prolonged thrombocytopenia after allogeneic marrow transplantation. Blood..

[8] Filipovich AH, Weisdorf D, Pavletic S, Socie G, Wingard JR, Lee SJ (2005). National Institutes of Health consensus development project on criteria for clinical trials in chronic graft-versus-host disease: I. Diagnosis and staging working group report. Biol Blood Marrow Transplant..

[9] Jagasia MH, Greinix HT, Arora M, Williams KM, Wolff D, Cowen EW (2015). National Institutes of Health Consensus Development Project on Criteria for Clinical Trials in Chronic Graft-versus-Host Disease: I. The 2014 Diagnosis and Staging Working Group report. Biol Blood Marrow Transplant..

[10] Inamoto Y, Flowers ME, Sandmaier BM, Aki SZ, Carpenter PA, Lee SJ (2014). Failure-free survival after initial systemic treatment of chronic graft-versus-host disease. Blood..

[11] Inamoto Y, Storer BE, Lee SJ, Carpenter PA, Sandmaier BM, Flowers ME (2013). Failure-free survival after second-line systemic treatment of chronic graft-versus-host disease. Blood..

